# Refeeding syndrome in small ruminants receiving parenteral nutrition

**DOI:** 10.1111/jvim.15840

**Published:** 2020-06-26

**Authors:** Daniela Luethy, Darko Stefanovski, Raymond W. Sweeney

**Affiliations:** ^1^ Department of Clinical Studies‐New Bolton Center (Luethy, Stefanovski, Sweeney), School of Veterinary Medicine University of Pennsylvania Kennett Square Pennsylvania USA

**Keywords:** hypophosphatemia, hypokalemia, hypomagnesemia, malnutrition

## Abstract

**Background:**

Small ruminants presented to tertiary care facilities commonly suffer from severe protein‐calorie malnutrition. Some of these patients require parenteral nutrition (PN; amino acids and dextrose with or without lipids) during hospitalization. Refeeding syndrome, a potentially fatal shift of electrolytes seen in malnourished patients during refeeding, may occur.

**Objective:**

(a) To report the prevalence of refeeding syndrome in small ruminants receiving PN and (b) to determine risk factors for the development of refeeding syndrome.

**Animals:**

Hospitalized small ruminants (n = 20) that received PN from 2010 to 2018 and that had serial (≥2) monitoring of serum electrolyte concentrations after initiation of PN.

**Methods:**

Retrospective case series. Refeeding syndrome was defined as the presence of at least 2 of the following electrolyte abnormalities after initiation of PN: hypophosphatemia, hypokalemia, hypomagnesemia, or some combination of these. Data was analyzed using Fisher's exact test, followed by univariate logistic regression.

**Results:**

Eleven of 20 (55%) animals met the definition of refeeding syndrome. Mean minimum serum phosphorus concentration in animals with refeeding syndrome was 1.96 ± 0.69 mg/dL (reference range, 4.2‐7.6 mg/dL). Eleven of 20 animals survived to discharge. Survival rate did not differ significantly between refeeding cases (4/11, 36.3%) and nonrefeeding cases (7/9, 77.8%; *P* = .09). Mean serum phosphorus concentration was significantly lower in nonsurvivors than in survivors (1.88 ± 0.10 mg/dL vs 4.32 ± 0.70 mg/dL, *P* = .006).

**Conclusions and Clinical Importance:**

We report the prevalence of refeeding syndrome in small ruminants receiving PN. Clinicians should anticipate refeeding syndrome after initiation of PN and consider pre‐emptive supplementation with phosphorus, potassium, magnesium, or some combination of these.

**Abbreviations**
ATP: adenosine triphosphate
PN: parenteral nutrition
RR: reference range
RER: resting energy requirement

## 1 INTRODUCTION

Small ruminants presented to tertiary care facilities often suffer from severe protein‐calorie malnutrition related to severe endoparasitism, catabolism associated with an underlying disease process, or poor animal husbandry. With the rising popularity of small ruminants as pets and as the focus of social media rescue groups, malnourished small ruminants are becoming more frequent patients seen in veterinary practice. Refeeding syndrome, a shift in electrolytes in malnourished patients being refed, is a possible complication of increased nutrition in these veterinary patients.^1^

Refeeding syndrome was first described in prisoners of war after World War II.^2^ The hallmark feature of refeeding syndrome is hypophosphatemia, although the syndrome involves changes in potassium, magnesium, sodium and fluid balance, thiamine deficiency, and changes in glucose, protein, and fat metabolism.^1^ In humans, refeeding syndrome most commonly is seen after prolonged starvation, in anorexia nervosa patients, patients with chronic alcoholism, obese patients undergoing massive weight loss, oncology patients undergoing chemotherapy, or in the refeeding of malnourished elderly patients.^3^, ^4^ Complications of refeeding syndrome are numerous and include electrolyte derangements, cardiac complications, and neurologic conditions.^3^, ^4^ The incidence of refeeding syndrome is highly variable depending on the study population. In a study of human internal medicine patients, 8% of the study population developed refeeding syndrome, whereas 54% of the patients were deemed to be at risk of developing refeeding syndrome.^5^

Little information is available in the literature regarding refeeding syndrome in veterinary species. Refeeding syndrome has been reported in case reports of cats.^6^, ^7^ In a study of dogs, researchers induced hypophosphatemia and neurologic abnormalities in starved dogs fed via intragastric catheter.^8^ In large animal species, limited information is available regarding refeeding syndrome. A retrospective study evaluating parenteral nutrition (PN) in alpacas reported refeeding syndrome in 3 of 21 alpacas.^9^ One study evaluating PN in neonatal foals reported complications including hypertriglyceridemia and catheter‐related complications, but no changes in serum electrolyte concentrations consistent with refeeding syndrome,^10^ whereas a retrospective study of 79 adult horses with gastrointestinal disease reported a single horse with hypomagnesemia and clinical signs suggestive of refeeding syndrome.^11^ Our objectives were to (a) report the prevalence of refeeding syndrome in small ruminants receiving PN and (b) identify risk factors for the development of refeeding syndrome.

## 2 MATERIALS AND METHODS

Cases were identified by an electronic medical record database search for goats and sheep that received PN (consisting of amino acids and dextrose with or without lipids) formulated to meet resting energy requirements (RER) while hospitalized at the George D Widener Hospital for Large Animals at the University of Pennsylvania's New Bolton Center during 2010 to 2018 and also had serial (≥2) monitoring of serum electrolyte concentrations (potassium, phosphorus, magnesium, calcium) after initiation of PN. Animals were excluded if they only received dextrose without amino acids or lipids, or if they received PN but serial serum electrolyte concentration monitoring was not performed. Information recorded for each patient included: signalment, history, presenting complaint, physical examination findings, clinicopathologic data, treatment, adverse events, long‐term outcome, and necropsy results (where applicable). The definition of refeeding syndrome was based on previous studies,^5^, ^9^ and required the presence of at least 2 of the following electrolyte abnormalities after initiation of PN: hypophosphatemia, hypokalemia, hypomagnesemia, or some combination of these (serum concentrations below the reference range [RR] on biochemical analysis after PN administration in a patient with a concentration that was initially within the RR).

### 2.1 Statistical analysis

Statistical analysis was performed using standard statistical software (Stata 15.1MP, StataCorp, College Station, Texas; WinSAAM, University of Pennsylvania, Kennett Square, Pennsylvania). Data were assessed for normality using the Shapiro‐Wilk test for normality. Categorical data were analyzed using Fisher's exact test to evaluate the association between survival and refeeding syndrome. For all independent variables, univariate logistic regression was performed to confirm the association with the outcomes of interest, survival and the occurrence of refeeding syndrome. All analyses were conducted using 2‐sided tests of hypotheses and a *P*‐value of <.05 was set as the criterion for statistical significance.

## 3 RESULTS

Thirteen goats and 7 sheep were included in the study, with a median age of 3 years (range, 2.5 months to 14 years). There were 10 females and 10 males. Eleven animals had a recorded body condition score of 1 of 5, whereas the remaining 9 did not have a body condition score recorded. Median body weight was 23 kg (range, 4.8‐95 kg). Final diagnoses (n) included severe endoparasitism (6), listeriosis (4), uterine torsion (1), paratuberculosis (1), generalized sepsis (1), necrotizing placentitis and pregnancy toxemia (1), esophageal dysfunction (1), enteritis and carpal subluxation (1), and undiagnosed (4).

Parenteral nutrition was prepared using a standard formula of 5 g/kg/day dextrose, 2 g/kg/day amino acids, and 1 g/kg/day lipids (n = 17) or 10 g/kg/day dextrose, 2 g/kg/day amino acids, and 1 g/kg/day lipids (n = 1), based on previously published formulas^12^ and at the discretion of the attending clinician. In 18 animals, these formulations provided 34 or 51 kcal/kg/day, respectively, and therefore provided a mean of 1.08 ± 0.23 × RER (range, 0.72‐1.5 × RER) based on the standard calculation of 70 × (body weight in kg)^0.75^. Two animals received PN consisting of dextrose and amino acids without lipids, formulated to provide 5 g/kg/day dextrose and 2 g/kg/day amino acids (25 kcal/kg/day), accounting for 1.06 × RER in these 2 animals. The target hourly flow rate was the total volume required to provide the above calculated amounts of nutrients divided by 24 hours. Parenteral nutrition was formulated aseptically under a laminar flow hood using a gravity system and consisted of a mixture of 50% dextrose solution (Covetrus, Nova‐Tech, Inc, Grand Island, Nebraska, USA), a 10% amino acid solution (10% Travasol Injection, Baxter Healthcare Corp, Deerfield, Illinois or 10% Aminosyn Injection, Hospira Inc, Lake Forest, Illinois), and a 20% lipid solution (Intralipid, Baxter Healthcare Corp, Deerfield, Illinois from 2010 to 2014 or Nutrilipid, B. Braun Medical Inc, Bethlehem, Pennsylvania from 2015 to 2018). All PN solutions contained a commercially available multivitamin solution (INFUVITE Adult Multiple Vitamins for Injection, Sandoz Canada Inc, Boucherville, Quebec, Canada). All animals received IV potassium chloride supplementation added to parenteral fluids at a concentration of 20 to 60 mEq/L. Parenteral nutrition was administered using an infusion pump via an aseptically‐placed 16 gauge polyurethane long‐term jugular catheter (Arrow central venous catheter kit 16 gauge single lumen, Teleflex, Inc, Morrisville, North Carolina) and dedicated line in all cases. Parenteral nutrition administration was initiated at 25% to 50% of the targeted RER and increased by 25% increments every 4 to 6 hours to the full target rate while monitoring blood glucose concentration. Cessation of PN administration was accomplished by 25% decrements every 4 to 6 hours while monitoring blood glucose concentration.

Median duration of hospitalization was 15 days (range, 6‐50 days). Median days to initiation of PN was 2 days (range, 0‐13 days). Median duration of PN administration was 6.5 days (range, 4‐18 days).

Eleven of 20 (55%) animals met the criteria for refeeding syndrome. Age, sex, and species were not significantly associated with development of refeeding syndrome. Of the animals that developed refeeding syndrome, 5 had concurrent hypophosphatemia, hypokalemia, and hypomagnesemia; 4 had concurrent hypophosphatemia and hypokalemia without hypomagnesemia; and 2 had concurrent hypophosphatemia and hypomagnesemia without hypokalemia. No animals that met the definition of refeeding syndrome lacked hypophosphatemia. Five of the 11 animals that had refeeding syndrome developed concurrent recumbency, weakness, or both at the time of their electrolyte abnormalities. Nine of 20 (45%) animals did not meet the criteria for refeeding syndrome. Of these 9, 2 developed hypophosphatemia but no other electrolyte abnormalities, 2 developed hypomagnesemia, and 1 developed hypokalemia. Table 1 summarizes serum electrolyte concentrations before and minimum concentrations after initiation of PN.

**TABLE 1:** Mean ± standard deviation electrolyte concentrations prior to and minimum concentration after initiation of parenteral nutrition (PN) in 20 small ruminants

	Mean phosphorus concentration (pre‐PN) (mg/dL)	Mean minimum phosphorus concentration (on PN) (mg/dL)	Mean change in phosphorus (mg/dL)	Mean potassium concentration (pre‐PN) (mmol/L)	Mean minimum potassium concentration (on PN) (mmol/L)	Mean change in potassium (mmol/L)	Mean magnesium concentration (pre‐PN) (mg/dL)	Mean minimum magnesium concentration (on PN) (mg/dL)	Mean change in magnesium (mg/dL)
All animals (n = 20)	5.56 ± 3.35	3.23 ± 2.11	−2.06 ± 3.88	4.26 ± 1.15	3.26 ± 0.66	−1.11 ± 1.39	2.09 ± 0.47	1.69 ± 0.39	−0.45 ± 0.81
Sheep (n = 7)	6.61 ± 4.54	2.77 ± 1.4	−3.38 ± 5.13	4.67 ± 1.58	3.32 ± 0.38	−1.83 ± 1.73	2.21 ± 0.64	1.65 ± 0.28	−0.8 ± 0.75
Goats (n = 13)	4.96 ± 2.46	3.47 ± 2.43	−1.11 ± 2.8	4.02 ± 0.8	3.24 ± 0.78	−0.72 ± 1.06	2.02 ± 0.33	1.72 ± 0.45	−0.25 ± 0.8
Refeeding syndrome (n = 11)	5.99 ± 4.15	1.96 ± 0.69	−3.49 ± 4.38	4.18 ± 1.23	2.88 ± 0.51	−1.3 ± 1.26	2.15 ± 0.58	1.63 ± 0.38	−0.33 ± 0.85
Nonrefeeding (n = 9)	5.08 ± 2.33	4.77 ± 2.26	−0.31 ± 2.35	4.36 ± 1.11	3.87 ± 0.31	−0.87 ± 1.59	2.03 ± 0.3	1.82 ± 0.41	−0.59 ± 0.79
Survivors (n = 11)	5.03 ± 1.98	4.32 ± 0.7	−0.71 ± 2.07	4.59 ± 1.44	3.63 ± 0.33	−1.21 ± 1.77	2.13 ± 0.46	1.78 ± 0.38	−0.65 ± 0.8
Nonsurvivors (n = 9)	6.29 ± 4.72	1.88 ± 0.1	−3.71 ± 4.97	3.88 ± 0.59	2.9 ± 0.71	−0.98 ± 0.8	2.05 ± 0.5	1.62 ± 0.41	−0.2 ± 0.8

*Note:* Change in electrolytes is the minimum electrolyte concentration minus the concentration prior to initiation to PN. Laboratory reference range for phosphorus concentration for sheep and goats were 4.0 to 8.9 and 4.2 to 7.6 mg/dL, respectively. Laboratory reference range for potassium concentration for sheep and goats were 4.1 to 5.8 and 3.6 to 4.8 mmol/L, respectively. Laboratory reference range for magnesium concentration for sheep and goats were 2.3 to 3.0 and 2.2 to 3.1 mg/dL, respectively.

During PN administration, all 20 animals received IV potassium chloride supplementation, 13/20 received IV phosphorus (as either potassium phosphate or sodium phosphate) supplementation, 8/20 received IV calcium gluconate supplementation, and 7/20 received IV magnesium sulfate supplementation. Of the 13 animals that received IV phosphorus supplementation, 4/13 were receiving IV phosphorus (0.3‐0.6 mmol phosphate/kg/day [28.5‐57 mg phosphorus/kg/day]) supplementation from the initiation of PN (before development of hypophosphatemia), all of which received an increase in phosphorus supplementation (0.6‐1.2 mmol phosphate/kg/day [57‐114 mg phosphorus/kg/day]) after identification of post‐PN hypophosphatemia. Nine of 13 received IV phosphorus supplementation after identification of post‐PN hypophosphatemia (0.6‐1.2 mmol phosphate/kg/day [57‐114 mg phosphorus/kg/day]). Of the 7 animals that received IV magnesium supplementation, 1 animal received IV magnesium supplementation from the initiation of PN (before development of hypomagnesemia), whereas the remaining 6 were treated in response to identification of post‐PN hypomagnesemia.

**FIGURE 1:**
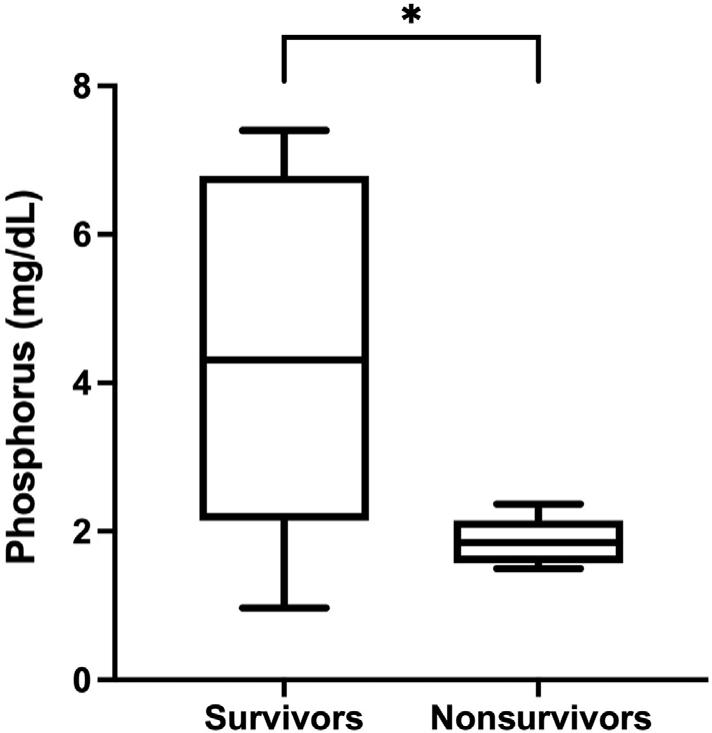
Minimum phosphorus concentration after initiation of parenteral nutrition in 20 small ruminants receiving parenteral nutrition. Line represents the mean, the box represents the interquartile range, and whiskers represent minimum and maximum values. Asterisk indicates mean phosphorus of nonsurvivors (n = 9, 1.88 ± 0.10 mg/dL) was significantly lower than for survivors (n = 11, 4.32 ± 0.70 mg/dL, *P* = 0.006, Fisher's exact)

Eleven of 20 (55%) animals survived to discharge. Survival rate was lower in refeeding syndrome cases (4/11, 36.3%) compared to nonfeeding syndrome cases (7/9, 77.8%), but the difference was not statistically significant (*P* = .09). Mean minimum post‐PN serum phosphorus concentration was lower in nonsurvivors than in survivors (1.88 ± 0.10 mg/dL vs 4.32 ± 0.70 mg/dL, *P* = .006; Figure 1). In the final univariate logistic regression analysis, however, the presence of post‐PN hypophosphatemia, hypokalemia or hypomagnesemia, as well as the minimum post‐PN serum concentrations of phosphorus, potassium and magnesium, were not associated with survival. The minimum post‐PN serum concentrations of phosphorus and potassium were significantly associated with refeeding syndrome in the univariate logistic regression (*P* = .009 and .012, respectively), but because these variables were included in the definition of refeeding syndrome, they were not considered clinically relevant. No differences between species or sex and survival were found. Age was associated with survival in the univariate logistic regression analysis, with younger animals more likely to survive than older animals. (*P* = .04; odds ratio, 0.79; 95% confidence interval, 0.63‐0.99).

## 4 DISCUSSION

Our retrospective study reports the prevalence of refeeding syndrome in small ruminants receiving PN. Approximately half of the animals in our study developed refeeding syndrome, indicating that clinicians should consider refeeding syndrome when initiating PN. Hypophosphatemia was a characteristic feature of refeeding syndrome in our study population, similar to studies in humans, and serum phosphorus concentration after initiation of PN predicted survival, but this association did not hold in the univariate logistic regression analysis. These findings improve our understanding of refeeding syndrome in small ruminants and may guide diagnosis and management of refeeding syndrome in clinical patients.

In chronic starvation, insulin concentrations decrease and glucagon concentrations increase, resulting in both gluconeogenesis and rapid conversion of glycogen stores to glucose, and ultimately resulting in the synthesis of glucose from the products of lipid and protein breakdown (fatty acids, glycerol, and amino acids). This results in catabolism and a loss of body mass.^4^ Over time, decreased PO intake of phosphorus occurs and intracellular phosphate is depleted as it is used for daily adenosine triphosphate (ATP) synthesis. During refeeding, metabolism shifts from fat to carbohydrate catabolism. Increased glucose (via enteral intake in monogastric animals or parenteral refeeding in all species) results in the release of insulin, which leads to increased cellular uptake of glucose, phosphate, potassium, magnesium, and water, as well as increased protein synthesis.^4^ This intracellular shift results in the electrolyte disturbances seen in refeeding syndrome and ultimately results in the clinical signs observed.

In both human and veterinary medicine, a single accepted definition of refeeding syndrome is lacking. In general, refeeding syndrome is defined as severe electrolyte shifts in malnourished patients undergoing enteral or parenteral refeeding.^4^ In human medicine, the incidence of refeeding syndrome varies in different studies, partly because of the lack of a universally accepted definition. One study found that 34% of human intensive care patients experienced refeeding hypophosphatemia.^13^ Another study evaluating all hospitalized human patients on artificial nutrition found an incidence of 1%.^14^ In veterinary medicine, limited information is available regarding the occurrence of refeeding syndrome. A number of reports in horses have evaluated PN, with most studies reporting no or few electrolyte abnormalities consistent with refeeding syndrome, regardless of age.^10^, ^11^ In retrospective studies of PN in horses, the most common findings were hypertriglyceridemia and catheter‐associated complications.^10^, ^11^, ^15^ Studies evaluating PN in calves similarly do not report the occurrence of refeeding syndrome.^16^ In a more recent study evaluating PN in nondomestic ruminants, 13 of 24 animals developed metabolic complications, including hypokalemia in 5 animals and hypophosphatemia in 3 animals.^17^ In that study, all animals that developed hypophosphatemia or hypokalemia died or were euthanized.

Notably, the small ruminants included in our study differ substantially with regard to their gastrointestinal physiology and carbohydrate metabolism as compared to monogastric species such as horses and humans, which make up the majority of literature regarding refeeding syndrome. It is not clear if refeeding syndrome would occur after enteral refeeding in ruminants, because PO glucose is not absorbed intact by the ruminant. However, in ruminant patients receiving IV glucose, it is not surprising that refeeding syndrome occurs similarly to monogastric animals. In addition, ruminants are unique in aspects of their phosphorus homeostasis. Phosphorus undergoes substantial salivary recycling in ruminants, which is essential for microbial digestion within the rumen. When phosphorus intake and stores are decreased, this salivary phosphorus recycling decreases and therefore ruminal function diminishes. Salivary recycling of phosphorus also may have substantial impact on serum phosphorus concentrations in inappetent ruminants, and may account for some of the hypophosphatemia seen in this study. Phosphorus absorption in ruminants is presumed to occur through similar mechanisms as in nonruminants, namely by vitamin D dependent active transport in the intestine when limited phosphorus is present in the diet, and by passive absorption when normal to large amounts of phosphorus are present in the diet. Unfortunately, vitamin D concentrations were not evaluated in our retrospective study, and no animals received vitamin D supplementation. Further evaluation of the causes and effects of refeeding syndrome in small ruminants is warranted in the future.

Although refeeding syndrome was common in our study, its association with survival did not reach statistical significance. Because of the small number of cases in our study it possibly was underpowered, and statistical significance may have been achieved with larger sample size. No risk factors or predictors for the development of refeeding syndrome were identified in our study. A study of humans evaluating acutely admitted internal medicine patients identified hypophosphatemia as the single key predictor of refeeding syndrome.^5^ In human medicine, oncology patients have been found to be at high risk. Other risk factors include acute or chronic malnutrition, alcohol abuse, old age, and malabsorption.^4^ Patients taking antacids or diuretics, which may be accompanied by loss of electrolytes via the gastrointestinal tract or kidneys, also have a higher risk of developing refeeding syndrome.^18^

In our study, nonsurvivors had a lower minimum serum phosphorus concentration after initiation of PN than did survivors, but an association between hypophosphatemia and nonsurvival was not found in the final univariate logistic regression analysis. Most of the animals reached their minimum serum phosphorus concentrations after 2 days of PN. Therefore, close monitoring of serum phosphorus concentration in the first few days of PN is indicated. All 11 animals with refeeding syndrome exhibited hypophosphatemia. In addition, 2 animals that did not meet the definition of refeeding syndrome also developed hypophosphatemia, emphasizing the importance of close monitoring of serum phosphorus concentration in any malnourished patient receiving refeeding (whether enterally or parenterally).

Limitations of our study include small sample size, lack of a universally accepted definition of refeeding syndrome, and the variability in PN administration, electrolyte supplementation, and disease states in the animals included in the study. In addition, dextrose alone can induce hypophosphatemia as well as induce hypokalemia and other electrolyte abnormalities. A previous study evaluating administration of 50% dextrose to healthy lactating dairy cows found that hypophosphatemia occurred in response to hyperglycemia or hyperinsulinemia after a continuous IV infusion of 50% dextrose, supporting the hypothesis of intracellular shifting of phosphorus in response to glucose.^19^ However, to limit variability among infusion rates of dextrose, we chose to include only animals receiving a standard formulation of PN. Administration of IV dextrose alone, however, also may induce changes in serum electrolyte concentrations and may lead to the development of refeeding syndrome in cachectic patients. In addition, clinical features of refeeding syndrome also may occur after enteral feeding,^4^ and the key prerequisite for development of refeeding syndrome is chronic nutritional deprivation.^4^ Additionally, not all animals received full RER via PN. Because RER is not linearly related to body weight and a standard formula that provides a standard number of kcal per day was used to calculate PN, the RER of animals under approximately 17 kg exceeded the 34 kcal/kg/day that was delivered via the standard PN formulation. Despite its limitations, our retrospective characterization of PN in hospitalized small ruminant patients is an important first step toward developing a standardized approach to PN in small ruminants.

In conclusion, refeeding syndrome occurs in approximately half of cachectic small ruminants receiving total or partial PN. Clinicians should anticipate refeeding syndrome after initiation of PN and consider pre‐emptive supplementation with phosphorus, potassium, magnesium, or some combination of these electrolytes.

## CONFLICT OF INTEREST DECLARATION

Authors declare no conflict of interest.

## OFF‐LABEL ANTIMICROBIAL DECLARATION

Authors declare no off‐label use of antimicrobials.

## INSTITUTIONAL ANIMAL CARE AND USE COMMITTEE (IACUC) OR OTHER APPROVAL DECLARATION

Authors declare no IACUC or other approval was needed.

## HUMAN ETHICS APPROVAL DECLARATION

Authors declare human ethics approval was not needed for this study.
